# Correction: How Long Is Too Long in Contemporary Peer Review? Perspectives from Authors Publishing in Conservation Biology Journals

**DOI:** 10.1371/journal.pone.0139783

**Published:** 2015-09-29

**Authors:** The PLOS ONE Staff

## Notice of Republication

This article was republished on September 18, 2015, because an incorrect version of the manuscript was uploaded following the final author revision. Please download this article again to view the correct version. The originally published, uncorrected article and the republished, corrected article are provided here for reference. In addition to changes made throughout the paper, please note that the Abstract has been updated to the following:

Delays in peer reviewed publication may have consequences for both assessment of scientific prowess in academia as well as communication of important information to the knowledge receptor community. We present an analysis on the perspectives of authors publishing in conservation biology journals regarding their opinions on the importance of speed in peer-review as well as how to improve review times. Authors were invited to take part in an online questionnaire, of which the data was subjected to both qualitative (open coding, categorizing) and quantitative analyses (generalized linear models). We received 637 responses to 6,547 e-mail invitations sent. Peer-review speed was generally perceived as slow, with authors experiencing a typical turnaround time of 14 weeks while their perceived optimal review time was six weeks. Male and younger respondents seem to have higher expectations of review speed than females and older respondents. The majority of participants attributed lengthy review times to reviewer and editor fatigue, while editor persistence and journal prestige were believed to speed up the review process. Negative consequences of lengthy review times were perceived to be greater for early career researchers and to have impact on author morale (e.g. motivation or frustration). Competition among colleagues was also of concern to respondents. Incentivizing peer-review was among the top suggested alterations to the system along with training graduate students in peer-review, increased editorial persistence, and changes to the norms of peer-review such as opening the peer-review process to the public. It is clear that authors surveyed in this study viewed the peer-review system as under stress and we encourage scientists and publishers to push the envelope for new peer-review models.

## Supporting Information

S1 File Originally published, uncorrected article. (PDF)

S2 File Republished, corrected article. (PDF)
