# Supplementary material for: How Long Is Too Long in Contemporary Peer Review? Perspectives from Authors Publishing in Conservation Biology Journals
Source: PLoS One. 2015 Aug 12;10(8):e0132557. doi: 10.1371/journal.pone.0132557 (PMC4533968; doi:10.1371/journal.pone.0132557)
Supplement: S1 File — (DOCX) [file pone.0132557.s001.docx]

**S1 Complete list of survey questions**

**How long is too long in contemporary peer-review?**

Questionnaire

*The following survey is aimed at obtaining your feedback on the peer-review process. The survey will take about 20 mins to complete. Your answers are critical to advancing our understanding of the dynamics of scientific peer-review and are much appreciated.*

*Many of the following questions focus on aspects relating to the duration of the review process (i.e. submission to decision).*

*Please restrict your responses based on research articles, review papers, meta-analyses, perspectives, and notes [Do NOT base response on letters, responses, critiques, and book reviews].*

*We recognize that the publication world has changed as we have moved away from paper submissions to e-submissions. For the purpose of this survey please focus on the last 5 years, where submissions and the entire review process are done electronically. Similarly, if you have only published one article in this timeframe, we ask that you do not participate in the survey at this time.*

*All information provided will be recorded completely anonymously.*

*Thank you for your cooperation and contribution to this research.*

**Warm-up! A few easy questions about the author.**

Here we would like to ask a few demographic questions. Reminder: all information is anonymous and remains confidential.

| 1. Do you work or have published in conservation biology or a closely related discipline? |
| --- |
| 1. What is your gender? |
| 1. What is your age group? |
| 1. Please describe your current employment: |
| 1. Please select the country you are currently based or work in: \| |
| 1. How many peer-reviewed articles have you published? |

**Section A: Review Metrics**

**For the below questions, please restrict your answers to the period between submission and the first editorial decision following formal peer-review. Please exclude instances where papers are rejected without being sent o referees.**

1. **What is the *slowest or longest* (in terms of length of time) review time that you have experienced as a first-author on a scientific paper (in weeks)?**

_______________________________

1. **What is the *fastest* or *shortest* (in terms of length of time) review time that you have experienced as a first author on a scientific paper (in weeks)?**

_______________________________

1. **In general, what do you consider a *typical* turnaround time for your submissions (in weeks)? (Defined as the duration from paper submission to when first decision is communicated to the first author)**

_______________________________

1. **In your opinion, what is a “slow” or “long” period for peer-review? Please specify in weeks:**
2. **In your opinion, what is a “fast” or “short” period for peer-review? Please specify in weeks:**
3. **In your opinion, what is the *optimal* review time that permits a thorough peer-review? Please specify in weeks:**
4. **In your opinion, does a “fast” peer review affect peer review quality?**
   1. **Yes, fast reviews have HIGHER review quality**
   2. **Yes, fast reviews have LOWER review quality**
   3. **No, “fast” and “slow” reviews have EQUAL quality**
   4. **No opinion**
5. **If a decision has not been made on your manuscript, how long will you wait until you make first contact with an editor/journal regarding the status of your “in review” manuscript? Please specify in weeks:**
6. _______
7. I never attempt to make contact with the editor/journal
8. **After a first status inquiry, will you make additional attempts to contact an editor/journal if time progresses without a response or decision?**
9. Yes
10. No
11. I never attempt to make contact with the editor/journal
12. **Have you ever threatened to, or actually have, withdrawn a submitted manuscript from a journal because you felt the review process was unsatisfactorily long?**
    1. Yes, I have withdrawn
    2. Yes, I have threatened to withdraw
    3. No

**If you answered “yes” for question 11, at what length of time was this deemed necessary?**

- 1. NA
  2. At this time point­­­­­­­­­­­­­­­­­­­­­­ _____

**­­­­­­**

1. **Do you think that when reviews are “long” that the paper is more likely to be:**
   1. Accepted
   2. Rejected
   3. Neither
   4. No opinion
2. **Do you think that when reviews are “short” that the paper is more likely to be:**

(a)Accepted

1. Rejected
2. Neither
3. No opinion

______________________

**Section B: The Author Experience**

1. **Do you feel that contacting an editor/journal regarding the status of a paper that is “in review” will influence their decision?**
2. Improve/Benefit
3. Jeopardize
4. No influence
5. No opinion
6. **In your opinion, who is *most* accountable for review speed?**
7. The editor
8. Journal staff
9. The referees/reviewers
10. Other (please specify ___)
11. **In your opinion, what are the ultimate factors that influence review speed (select all that apply)? (Likert Scale question) Greatly slows review speed, Somewhat slows reviews speed, No impact, Somewhat speeds up review, Greatly speeds up review:**
    1. Scientific significance for advancing the field of study
    2. Conservation implications of results
    3. Policy implications of results
    4. Potential public interest of potential for media attention
    5. Length of paper
    6. Journal prestige or impact factor
    7. Maximum ‘allocated’ review times for each journal
    8. Persistence of editorial team
    9. Number of reviewers
    10. Editor fatigue (lack of time, etc)
    11. Reviewer fatigue (lack of time, etc.)
    12. Other ________________(please specify)
12. **Is review duration an important factor for you when considering which journal to submit your research to?**
13. Yes
14. Sometimes
15. Never
16. No opinion
17. **After experiencing a “long” review period, I am:**
18. Less likely to submit to that journal again relative to other journals
19. More likely to submit to that journal again relative to other journals
20. Neither more nor less likely to submit to that journal again
21. Depends on journal impact factor or prestige
22. No opinion
23. **After experiencing a “short” review period, I am:**
24. Less likely to submit to that journal again relative to other journals
25. More likely to submit to that journal again relative to other journals
26. Neither more nor less likely to submit to that journal again
27. Depends on journal impact factor or prestige
28. No opinion
29. **Do you ever discuss speed of review with your colleagues?**
30. Daily
31. Weekly
32. Monthly
33. Once a year
34. Never
35. **Have you ever “blacklisted” a journal (i.e. chosen not to resubmit manuscripts to that journal in the future) due to their speed of review?**

1. Yes
2. No
3. I prefer not to answer
4. **Do you feel a lengthy peer review process affects your career?**
   1. **Yes**
   2. **No**

**If you answered “yes” to question 22, please provide** examples if you have had specific **Response: ____**

1. **Do you believe there are consequences of manuscript review duration for our fiend in Conservation Science?**
   1. Yes
   2. No
   3. Provide examples if you have them
2. **Do you think the review process should be altered to change review time?**
   1. Yes
   2. No
   3. No opinion
   4. Prefer not to answer
   5. If Yes, explain how? _________________________
3. **Does publishing in a top-tier, high impact journal, justify a rapid or delayed review time?**
4. Yes
5. No
6. No opinion
7. I prefer not to answer
8. If Yes, which situation is justified:
   1. Rapid
   2. Delay
   3. Both
   4. Neither
   5. I prefer not to answer
9. **Does publishing in a low-tier, low impact journal, justify a rapid or delayed review time?**
10. Yes
11. No
12. No opinion
13. I prefer not to answer
14. If Yes, which situation is justified:
    1. Rapid
    2. Delay
    3. Both
    4. Neither
    5. I prefer not to answer
15. **Do you think the review process should be altered if the results have high scientific significance for advancing the field of study?**
    1. Yes
    2. No
    3. Neither
    4. No opinion
    5. Prefer not to answer

**If you answered *YES* for question 26, should it be:**

- 1. Faster?
  2. Slower?

1. **Do you think the review process should be altered if the results have high conservation implications?**
   1. Yes
   2. No
   3. Neither
   4. No opinion
   5. Prefer not to answer

**If you answered *YES* for question 31, should it be:**

- 1. Faster?
  2. Slower?

1. **Are you aware of any instances in which a “lengthy” peer review had marked effects on a conservation problem/issue? If yes, please describe.**
   1. No
   2. Yes­­­, ­____
2. **Do you think the review process should be altered if the results have high policy implications?**
   1. Yes
   2. No
   3. Neither
   4. No opinion
   5. Prefer not to answer

**If you answered *YES* for question 29, should it be:**

- 1. Faster?
  2. Slower?

1. **Do you think the review process should be altered if the results have the potential for high public interest or media attention?**
   1. Yes
   2. No
   3. Neither
   4. No opinion
   5. Prefer not to answer
2. **Do you think that open-access journal in which authors must pay a fee to publish should have higher quality of ‘customer service’ than other publishing options, such as faster review and publication times?**
   1. Strongly agree
   2. Agree
   3. Neither agree nor disagree
   4. Disagree
   5. Strongly disagree

**Section C: Author Publishing History**

You’re almost done! Just a wrap up and a few characteristic about the author’s publishing history.

1. **Approximately how many peer-reviewed scientific publications do you publish per year (first author and co-author)?**
2. 3 or less
3. Between 3-5
4. Between 5-7
5. Between 5-9
6. Greater than 9
7. **What year did you publish your first scientific publication (first author OR co-author)?**

**_________________**

1. **Have you served as a referee for a peer-reviewed journal?**
   1. Yes
   2. No
2. **How often do you serve as referee for a peer-reviewed journal per year?**
   1. 1-5
   2. 5-10
   3. 10-15
   4. >15
3. **As a referee, how quickly on average do you review manuscripts when assigned? Please specify in weeks:**
4. **As a referee, what is the average length of time it takes for you to review a manuscript (from first read until submission)?**
5. **As a referee, are you more likely to review a manuscript more quickly for a “higher-tier” versus “lower-tier” journal, even when given equal amounts of time for review?**
   1. **Yes**
   2. **No**

**You have reached the end of the survey! Please leave any additional comments here:**
