# Supplementary material for: How Long Is Too Long in Contemporary Peer Review? Perspectives from Authors Publishing in Conservation Biology Journals
Source: PLoS One. 2015 Aug 12;10(8):e0132557. doi: 10.1371/journal.pone.0132557 (PMC4533968; doi:10.1371/journal.pone.0132557)
Supplement: S2 File — (DOCX) [file pone.0132557.s002.docx]

S2: GLM Data Analysis Supplement for Models 1-3

Data Analysis

Categorical and continuous covariates were first examined using Cleveland dotplots, correlation plots, and boxplots. Covariates included: age category (AGE), gender (GEN), number of publications (NP), experience regarding review period (EXP, # of weeks, depending on question). The response variables were based on a respondent’s opinion, i.e., their opinion or expectation regarding the review period (# of weeks). These response variables included opinions related to a respondent’s “optimal” review period (OOR, Model 1), opinion on a “short” review period (OSR, Model 2), and opinion on a “long” review period (OLR, Model 3). The covariate “Experience” related to a respondent’s experienced “typical” review period (EXP, Model 1), experienced “short” review period (EXP, Model 2), or experienced “long” review period (EXP, Model 3). The categories for country of employment or employment type were not included in the analyses due to the large number of countries (n>50) and highly uneven number of respondents for each. Influential observations were removed from the data. Such extreme values constituted less than 1% of the data available for each analysis. Given a low number of respondents, the age category of “over 65” and the gender category “prefer not to say” were removed from the analyses. Generalized linear models (GLM) were used to model the data following the standard approaches to analyze count data (e.g., McCullagh and Nelder 1989). To model OOR and OSR, *Y_i_* represents the opinion (i.e., expectation) on review time, in number of weeks, from the *i*^th^ respondent. The *Y_i_* are assumed to follow a Poisson distribution with mean *μ_i_*. The expected counts were related to a full set of explanatory variables by modelling the counts, *Y_i_*, assuming Poisson errors and a log link. Due to high variability in the residuals (i.e., overdispersion), for the model of OLR, *Y_i_* was assumed to follow a negative binomial distribution with a log link between the mean and the predictor function (Zuur et al. 2009). Full models contained the terms: age category (AGE), gender (GEN), number of publications (NP), experience (EXP), GEN:AGE, GEN:NP, EXP:GEN, EXP:NP, and EXP:AGE. Backwards model selection was applied using the drop1 command, which drops one explanatory variable, beginning with interactions, and each time applies an analysis of deviance test (Chambers 1992; Zuur et al. 2009). The null hypothesis assumes no difference between the deviances, thus a large difference in deviance is evidence against the null hypothesis. Model selection is conducted by an iterative process where the least significant term is removed and the model refitted until all remaining terms are significant (Zuur et al. 2009). Final models were checked for overdispersion of the residuals and validated by plotting the Pearson and deviance residuals against all covariates, including those not retained in the final model.

**Final fitted models**

Model 1 - Opinion on the optimal review period

$$Y_{i} \sim Poisson \left( \mu_{i} \right)$$

$E(Y_{i})$ = $\mu_{i}$ and var($Y_{i})= \mu_{i}$

$$\log\left( \mu_{i} \right)= \propto+ B_{1}{\times EXP}_{i}+\beta_{2}\times{GEN}_{i}+\beta_{3}\times{EXP}_{i}\times{GEN}_{i}+ \varepsilon_{i}$$

$$\varepsilon_{i}\sim N(0, \sigma_{\varepsilon}^{2})$$

Where $Y_{i}$ is opinion, in number of weeks, on what constitutes an optimal review period according to respondent i. $Y_{i}$ is assumed to be Poisson distributed with a mean of $\mu_{i}$. The expected mean and variance are equal and given by E and var respectively. The error $\varepsilon_{i}$is assumed to be normally distributed with a mean zero and variance$\sigma^{2}$. ${EXP}_{i}$ is the experienced typical review period by respondent i.

Model 2 - Opinion on a short review period

$$Y_{i} \sim Poisson \left( \mu_{i} \right)$$

$E(Y_{i})$ = $\mu_{i}$ and var($Y_{i})= \mu_{i}$

$$\log\left( \mu_{i} \right)= \propto+ B_{1}{\times EXP}_{i}+\beta_{2}\times{AGE}_{i}+\beta_{3}\times{GEN}_{i}+\beta_{4}\times{GEN}_{i}\times{AGE}_{i}+\beta_{5}\times{EXP}_{i}\times{GEN}_{i}+\beta_{6}\times{EXP}_{i}\times{AGE}_{i}+ \varepsilon_{i}$$

$$\varepsilon_{i}\sim N(0, \sigma_{\varepsilon}^{2})$$

Where $Y_{i}$ is opinion, in number of weeks, on what constitutes a short review period according to respondent i. $Y_{i}$ is assumed to be Poisson distributed with a mean of $\mu_{i}$. The expected mean and variance are equal and given by E and var respectively. The error $\varepsilon_{i}$is assumed to be normally distributed with a mean zero and variance$\sigma^{2}$. ${EXP}_{i}$ is the experienced short review period by respondent i.

Model 3 - Opinion on a long review period

$$Y_{i} \sim NB \left( \mu_{i},k \right)$$

$E(Y_{i})$ = $\mu_{i}$ and var($Y_{i})= \mu_{i}+ \frac{\mu_{i}^{2}}{k}$

$$\mu_{i}= e^{\propto+ B_{1}{\times EXP}_{i}+\beta_{2}\times{GEN}_{i}}$$

Where $Y_{i}$ is opinion, in number of weeks, on what constitutes a long review period according to respondent i. $Y_{i}$ is assumed to have a negative binomial distribution with a mean of $\mu_{i}$ and dispersion parameter *k*. The expected mean and variance are equal and given by E and var respectively. ${EXP}_{i}$ is the experienced long review period by respondent i.

References

McCullagh, P., and Nelder, J.A. 1989. Generalized Linear Models, 2^nd^ Ed. Chapman and Hall, London.

Chambers, J. M. 1992. Linear models. Chapter 4 of Statistical Models in S eds J. M. Chambers and T. J. Hastie, Wadsworth & Brooks/Cole.

Zuur, A. F., E. N. Ieno, N. Walker, A. A. Saveliev, and G. M. Smith. 2009. Mixed effects models and extensions in ecology with R. New York: Springer.
