# Supplementary material for: Correction: How Long Is Too Long in Contemporary Peer Review? Perspectives from Authors Publishing in Conservation Biology Journals
Source: PLoS One. 2015 Sep 29;10(9):e0139783. doi: 10.1371/journal.pone.0139783 (PMC4587732; doi:10.1371/journal.pone.0139783)
Supplement: S1 File — (PDF) [file pone.0139783.s001.pdf]

PLOS ONE

RESEARCH ARTICLE

How Long Is Too Long in Contemporary Peer Review? Perspectives from Authors Publishing in Conservation Biology Journals

Vivian M. Nguyen[1]*, **Neal R. Haddaway**[2], **Lee F. G. Gutowsky**[1], **Alexander D. M. Wilson**[1], **Austin J. Gallagher**[3,4], **Michael R. Donaldson**[1], **Neil Hammerschlag**[3], **Steven J. Cooke**[1,5]

1 Fish Ecology and Conservation Physiology Laboratory, Department of Biology, Carleton University, Ottawa, Ontario, Canada, **2** MISTRA EviEM, Royal Swedish Academy of Sciences, Stockholm, Sweden, **3** Rosenstiel School of Marline and Atmospheric Science, University of Miami, Miami, FL, United States of America, **4** Beneath the Waves, Inc., Syracuse, NY, United States of America, **5** Institute of Environmental Science, Carleton University, Ottawa, Ontario, Canada

* Vivian.nguyen@carleton.ca

CrossMark
click for updates

OPEN ACCESS

Citation: Nguyen VM, Haddaway NR, Gutowsky LFG, Wilson ADM, Gallagher AJ, Donaldson MR, et al. (2015) How Long Is Too Long in Contemporary Peer Review? Perspectives from Authors Publishing in Conservation Biology Journals. PLoS ONE 10(8): e0132557. doi:10.1371/journal.pone.0132557

Editor: Miguel A Andrade-Navarro, Johannes-Gutenberg University of Mainz, GERMANY

Received: March 1, 2015

Accepted: June 16, 2015

Published: August 12, 2015

Copyright: © 2015 Nguyen et al. This is an open access article distributed under the terms of the Creative Commons Attribution License, which permits unrestricted use, distribution, and reproduction in any medium, provided the original author and source are credited.

Data Availability Statement: Data are available in the paper and supporting information files.

Funding: This work was supported by the Natural Sciences and Engineering Research Council, 315918-166, http://www.nserc-crsng.gc.ca/index_eng.asp and the Canada Research Chair, 320517-166, http://www.chairs-chaires.gc.ca/home-accueil-eng.aspx. The funders had no role in study design, data collection and analysis, decision to publish, or preparation of the manuscript.

Abstract

Delays in peer reviewed publication may have consequences for both assessment of scientific prowess in academics as well as communication of important information to the knowledge receptor community. We present an analysis on the perspectives of authors publishing in conservation biology journals regarding their opinions on the importance of speed in peer-review as well as how to improve review times. Authors were invited to take part in an online questionnaire, of which the data was subjected to both qualitative (open coding, categorizing) and quantitative analyses (generalized linear models). We received 637 responses to a total of 6,547 e-mail invitations sent. Peer-review speed was generally perceived as slow, with authors experiencing a typical turnaround time of 14 weeks while their perceived optimal review time is six weeks. Male and younger respondents seem to have higher expectations of review speed than females and older respondents. Majority of participants attributed lengthy review times to the 'stress' on the peer-review system (i.e., reviewer and editor fatigue), while editor persistence and journal prestige were believed to speed up the review process. Negative consequences of lengthy review times appear to be greater for early career researchers and can also have impact on author morale (e.g. motivation or frustration). Competition among colleagues were also of concern to respondents. Incentivizing peer review was among the top suggested alterations to the system along with training graduate students in peer review, increased editorial persistence, and changes to the norms of peer-review such as opening the peer-review process to the public. It is clear that authors surveyed in this study view the peer-review system as under stress and we encourage scientists and publishers to push the envelope for new peer review models.

Competing Interests: The authors have declared that no competing interests exist.

Introduction

Peer reviewed publications remain the cornerstone of the scientific world [1, 2] despite the fact that the review process is not infallible [3, 4]. Such publications are an essential means of disseminating scientific information through credible and accessible channels. Moreover, academic institutions evaluate scientists based on the quantity and quality of their research via publication output. Given the importance of peer-review to the dissemination of information and to the researchers themselves, it is of little surprise that the process of scientific publishing has been a subject of discussion itself. For example, researchers have explored the many and various biases associated with contemporary peer-review [e.g., gender [5], nationality/language [6], and presence of a "known" name/academic age [7]], with a goal of improving the objectivity, fairness and rigor of the review process [8]. What has received less attention is the extent to which the peer review process causes detriment through overly slow dissemination of timely science-based conservation strategies in an era of human-induced rapid environmental change (HIREC; [9]) in the Anthropocene [10]. Given the significance of publications for science and evidence-based conservation [11], efforts to improve the peer-review system are warranted to ensure that delays in publication do not have significant impacts on the transition of scientific evidence into policy.

Despite the switch from surface mail to online communication channels and article submission [12, 13], review processes may still stretch into months or even years. Such extreme delays have consequences for both the assessment of scientific prowess (e.g., tenure, employment, promotion) in academics and also delay the communication of important information for threatened habitats or species. Presumably having "rapid" turnaround times is desirable for authors [14], particularly early career researchers [15], but also puts "stress" on the peer-review system. Although review time certainly is discussed informally, there is very little known about what authors themselves think about the speed of peer-review and how it could be improved. For example, what is an acceptable timeline for a review? How long should authors wait before contacting editors about the progress of a review? What do authors perceive as trade-offs in quality versus speed of a review? What strategies can an author use to try to elicit a more rapid review process? What are the underlying factors that influence variation in review time? Do author demographics play a role in the perspective in the variation of review time? Finally, what does a "long" review mean to career development, scientific progress, and the future behavior of authors with respect to selecting potential publishing outlets? These questions might seem obvious or inherent given our publishing roles and requirements as active researchers, but they have yet to be addressed formally in the scientific literature.

Here we present an analysis on the perspectives about the speed and importance of review times among a subset of authors of papers within the realm of "conservation biology." Conservation biology is a field with particular urgency [16] for evidence to inform decisions but has not received as much attention on its peer-review system as other urgent fields such as health and medical sciences [17, 18]. We discuss the findings as they relate to peer review duration and present author perspective on how to improve the speed of peer-review.

Methods

Data Collection and Sampling

We extracted the e-mail addresses of authors that published in the field of "conservation biology" from citation records within the Web of Science online database. A search was undertaken on 9 April, 2014 using Web of Science [consisting of Web of Science Core Collections, Biosis Previews (subscription up to 2008), MEDLINE, SciELo and Zoological Record]. We used the

following search string, and limited the search to 2013 (to ensure all authors were still active): "conservation AND *diversity". Search results were refined to include entries for the following Web of Science subject categories alone: environmental sciences ecology, biodiversity conservation, zoology, plant sciences, marine freshwater biology, agriculture, forestry, entomology, fisheries. A total of 6,142 results were obtained, where 4,606 individual e-mail addresses were extracted. E-mails were sent to this mailing list inviting authors to participate in an anonymous online questionnaire hosted on Fluid Surveys; however, of these e-mails, 312 addresses were non-functional or no longer active. Individuals with e-mails that bounced back indicating a change of e-mail, were sent an invitation to the new e-mail address indicated. We sent an additional invitation on 22 May, 2014 using a mailing list produced from an additional extraction of 2,679 e-mail addresses obtained from another search using the above string and subject categories but restricted to 2012, with 426 addresses that were non-functional or no longer active. Reminders were sent to all e-mail addresses between 18–20 June, 2014, and closed access to the online questionnaire on 3 July, 2014.

Survey Instrument

The entire questionnaire was composed of 38 open- and closed-ended questions, of which a subset of the questions relevant to review times were used for this study. We asked respondents to focus their experiences in the last five years, given the major phase shift in review protocols in earlier years associated with the move to electronic-based communication [19, 20]. However, we did anticipate observing different responses between those that were active in publishing in the pre-electronic era and those that have only published since electronic submission and review became standard practice. While it is not possible to decouple author age/career stage as a potential response driver in the questionnaire [15], we nonetheless explored the association between time since first peer-reviewed publication and author responses. The questionnaire began with questions that assessed the participants' opinions on various "review metrics" (e.g., opinions of slow vs. rapid review durations, optimal review duration—see supplemental information for full survey questions (S1 File). This section was followed by questions associated with the respondent's experience and expectations as an author, and their potential behaviour with respect to lengthy review times. Additionally, we assessed participants' perspective on factors that ultimately influence review speed using open-ended questions and Likert type questions. We then asked whether the peer-review system should be altered and how it should be altered. Lastly, we recorded respondent characteristics such as socio-demographic information, publishing experience and frequency, as well as other experiences with the peer-review system (e.g. referee experience). It is important to note that there could be potential inaccuracies in perceptions of time and events due to self-reporting and recall bias, when someone may perceive a length of time to be quicker or slower than it is in reality. All but author characteristic questions in the survey were optional, and the number of responses (the sample size, n) therefore varies accordingly at or below the total number of respondents. The questionnaire was pre-tested with five authors, and protocols were approved by Carleton University Research Ethics Board (100958).

Data Analysis

For open-ended responses, we categorized the data by common themes that emerged among responses (i.e. open coding) [21] using QSR NVivo 10. We use narrative-style quotes from the responses throughout the paper to illustrate the details and properties of each category or theme. We quantified certain responses using frequency counts of the coded themes to provide proportions of respondents that agree with an idea/theme or to provide a number of responses

that corresponded with a theme. For the purpose of article clarity and conciseness, we report the majority of responses in percentage and chose to omit reporting the remainder of the responses when they are responses of no opinions or neutrality (e.g., when a respondent responds to a choice as "neither").

Generalized linear models were used to identify how demographic information (e.g., gender), career status (e.g., number of publications), and experience regarding review times (# of weeks for either a "typical" (TYP), "short" (ES), or "long" (LS) review period) explained respondents' expectations (i.e., opinion) for the length of time that constitutes an optimal (Model 1), short (Model 2) and long review time (Model 3). Response variables (modeled as # of weeks) were assumed to follow a Poisson or negative binomial distribution (i.e., when residuals were overdispersed) with normally distributed errors. The best model to explain respondent opinion was selected using backwards model selection [22, 23]. Details on the statistical methods and the results are found in (S2 File).

Results and Discussion

Response rate and overall respondent characteristics

We received 673 responses out of all the invited participants (N = 6,547), of which 461 completed the questionnaire to the end, with the possibility of skipping some questions (see S3 File for raw data). The remainder of participants partially completed the questionnaire, thus the number of responses varied by question. Of all the respondents, 28% were female and 63% were male (<1% preferred not to say). This may lead to a slight male-dominant perspective in our results. It also appears that respondents in our study are largely well into their careers. Most respondents ranged between 31–40 years old (38.2%), followed by 41–50 years (24%), 51–64 years (18%), 21–30 years (11%), less than 5% of respondents were 65 years or older, and <1% were under 21 years old (2 respondents).

Overall, responses came from 119 different countries. We then categorized the countries based on economic income set out by the World Bank (2014). The majority of respondents (N = 640) worked in countries of high-income economies (78%), followed by upper-middle-income economies (17%), lower-middle-income (4%), and less than 2% for low-income economies. The top countries participating in this study included the United States (17%), the United Kingdom (10%), Australia (8%) and Brazil (7%). The majority of respondents (N = 611) were from academia (77% of which 15% were graduate students), governmental or state agencies (11%), non-government or non-profit organizations (10%), and the private sector (2%) which can include consulting and non-academic research institutes among others. The participant characterizing suggests that the author perspectives in this article are largely bias towards industrialized nations and academia.

Author publishing and referee experiences

A larger proportion of participants published their first paper within the last decade (44% of 451 respondents published in 2000–2009, and 19% published in 2010 and after), which indicates a bias toward authors that are potentially in their mid-careers. About half of the respondents have published > 20 publications (with 21% of 623 respondents publishing >50), and only 10% have published <10. Half of the participants publish < 3 papers per year, 35% publish 4–6, 10% publish 7–10, and only 3% of participants publish >10 papers per year. Furthermore, nearly half of the respondents act as journal referees 1–5 times per year (48% of N = 450). Twenty percent of respondents are highly active referees (reviewing manuscripts >10 times per year), 25% being referees 6–10 times, and < 10% reviewing manuscripts only once a year. Overall, the majority of respondents have been publishing for at least 10 years and

at least half of them are highly experienced with the peer-review process as both authors and referees. As such, the perspectives gathered in our questionnaire come from highly experienced authors that are actively publishing and therefore familiar with the peer-review system.

Peer review duration: experiences and expectations

We asked participating authors about their experience with peer-review durations (i.e. period between initial submission and first editorial decision following formal peer review), and 368 respondents gave useable/ complete answers. The average (mean ± SD) shortest or quickest review time was reported to be 5.1 ± 6.0 weeks (Table 1), while the opinion of a "fast" review period was on average 4.4 ± 2.9 weeks. While the opinion of a "slow" review period was on average 14.4 ± 8.2 weeks, the longest or slowest review time was reported on average to be 31.5 ± 23.8 weeks (Table 1)—nearly double what the respondents perceive as slow. Furthermore, respondents reported that a "typical" turnaround time for a manuscript submission was on average 14.4 ± 6.0 weeks (ranging between 2–52 weeks), and that the optimal review period on average (median) is 6.4 ± 4 weeks. An optimal range for peer review durations were 1–20 weeks with majority falling within eight weeks or under (86% of 366 responses).

The fact that respondent opinions and actual experiences of short or long review durations are not aligned, and that their experiences of review durations are lengthier (nearly double the "optimal" time), indicate that the overall perception of the peer-review system is slow. Results reported here may provide indicators for conservation biology related journals to gauge their performance on review time and improve author experiences and satisfaction. In a broad review (over 4000 respondents from across disciplines), Mulligan et al. [24] noted that 43% of respondents felt that the time it took to the first decision for their last article was slow or very slow. Mulligan et al. [24] asked authors about whether their last manuscript review (to first decision) took longer than 6 months and reported a mean of 31% but noted some differences among disciplines. For example, reviews in the physical sciences and chemistry rarely (15%) take longer than 6 months while those in the humanities, social science and economics were more likely to take longer than 6 months (i.e., 59%). Mulligan et al. [24] included a category called "agricultural and biological sciences" and reported 29% of respondents indicated reviews took longer than 6 months with 45% reporting 3 to 6 months. In general, these findings are consistent with the responses we obtained from a focused survey of scientists working in conservation biology.

Respondents did not perceive "fast" or "slow" reviews to influence review quality (75% of 547 useable responses), with the exception of 8% of respondents who believed that fast reviews have higher review quality and another 8% believed fast reviews have lower review quality (10% had no opinion). Therefore, faster review times should presumably be beneficial to the

Table 1. Reported opinions and experiences of peer-review durations.

Category	Average time in weeks (mean ± SD*)	Range (weeks)
Shortest or quickest review time reported	5.1 ± 6.0	1–88
Opinion of fast review time	4.4± 2.9	1–26
Longest or slowest review time reported	31.5 ± 23.8	1–200
Opinion of slow review time	14.4 ± 8.2	1–100
Typical turnaround time reported	14.4 ± 6.0	1–54
Opinion of optimal review time	6.4 ± 4	1–52

*SD = Standard Deviation

doi:10.1371/journal.pone.0132557.t001

authors, the journals and the relevant field given the belief that review speed does not affect quality, although this has not been tested empirically. We discuss mechanisms to improve review times based on this information later in this article.

Who expects what in peer review duration?

A respondent's opinion for an optimal review time depended on a two-way interaction between respondent experience and gender (TYP*Gender, L- Ratio Test = 5.9, df = 1, P = 0.015). According to both male and female respondents, the optimal length of time for a review should always be shorter than what they have experienced as "typical" (Fig 1). Males publishing in conservation biology believe that the optimal review period should remain relatively short, despite what they have experienced as "typical". The opinion or expectation of female respondents is more closely related to their experience (Fig 1). For instance, female

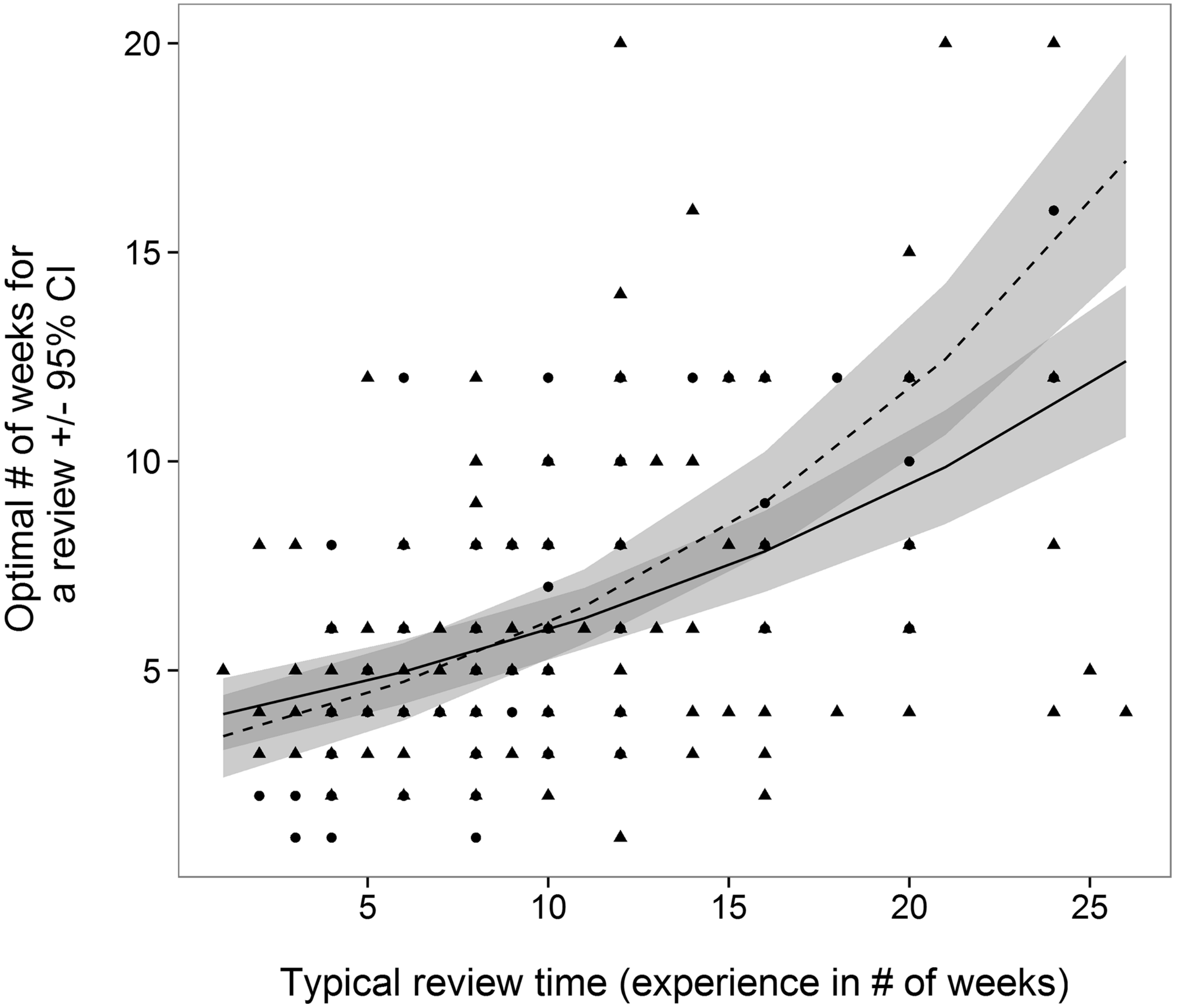

Fig 1. Male (solid line) and female (dashed line) researcher opinion on the optimal number of weeks ± 95 CI for the review process given their experience (# of weeks for a "typical" review).

doi:10.1371/journal.pone.0132557.g001

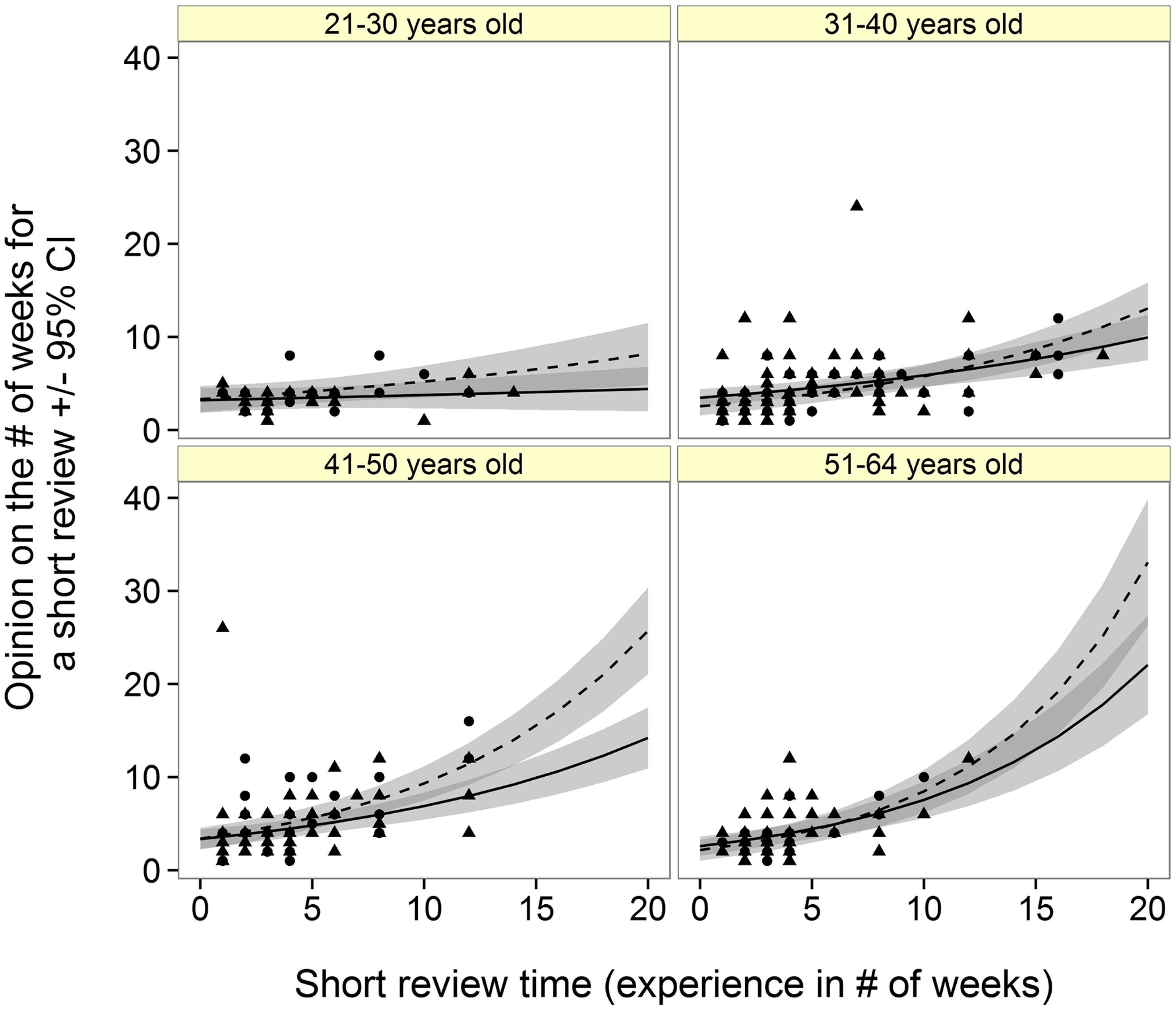

Fig 2. Male (solid line) and female (dashed line) researcher opinion on the time (# weeks ± 95% CI) considered for a short review turn around given their experience (# of weeks for a short review) and age category.

doi:10.1371/journal.pone.0132557.g002

researchers suggest the optimal review period to be 34 weeks when they stated a typical review period was 36 weeks; a difference of only 2 weeks. On the other hand, male researchers expect reviews to be returned 16 weeks earlier when they stated a typical review period takes 36 weeks; a difference of 20 weeks. Opinion on what constitutes a short review period (Model 2) was dependent on several two-way interactions including: Age*Gender (L- Ratio Test = 10.6, df = 3, P = 0.01), ES*Gender (L- Ratio Test = 5.1, df = 1, P = 0.02), ES*Age (L- Ratio Test = 11.5, df = 3, P = 0.01). For respondents over 41 years old, experience and opinion are more closely related than compared to younger respondents who suggest a short review is $\leq$ 10 weeks, regardless of experience (Fig 1). Similar to the trend in Model 1, female experience and opinion were more closely matched than males, however this was evident for respondents 41–50 years old (Fig 2). Finally, opinion on a long review period (Model 3) was dependent on LS (L- Ratio Test = 61.7, df = 1, P $<$ 0.001) and Gender (L- Ratio Test = 6.0, df = 1, P = 0.01). Here, respondents always expected "long" review periods to be many weeks

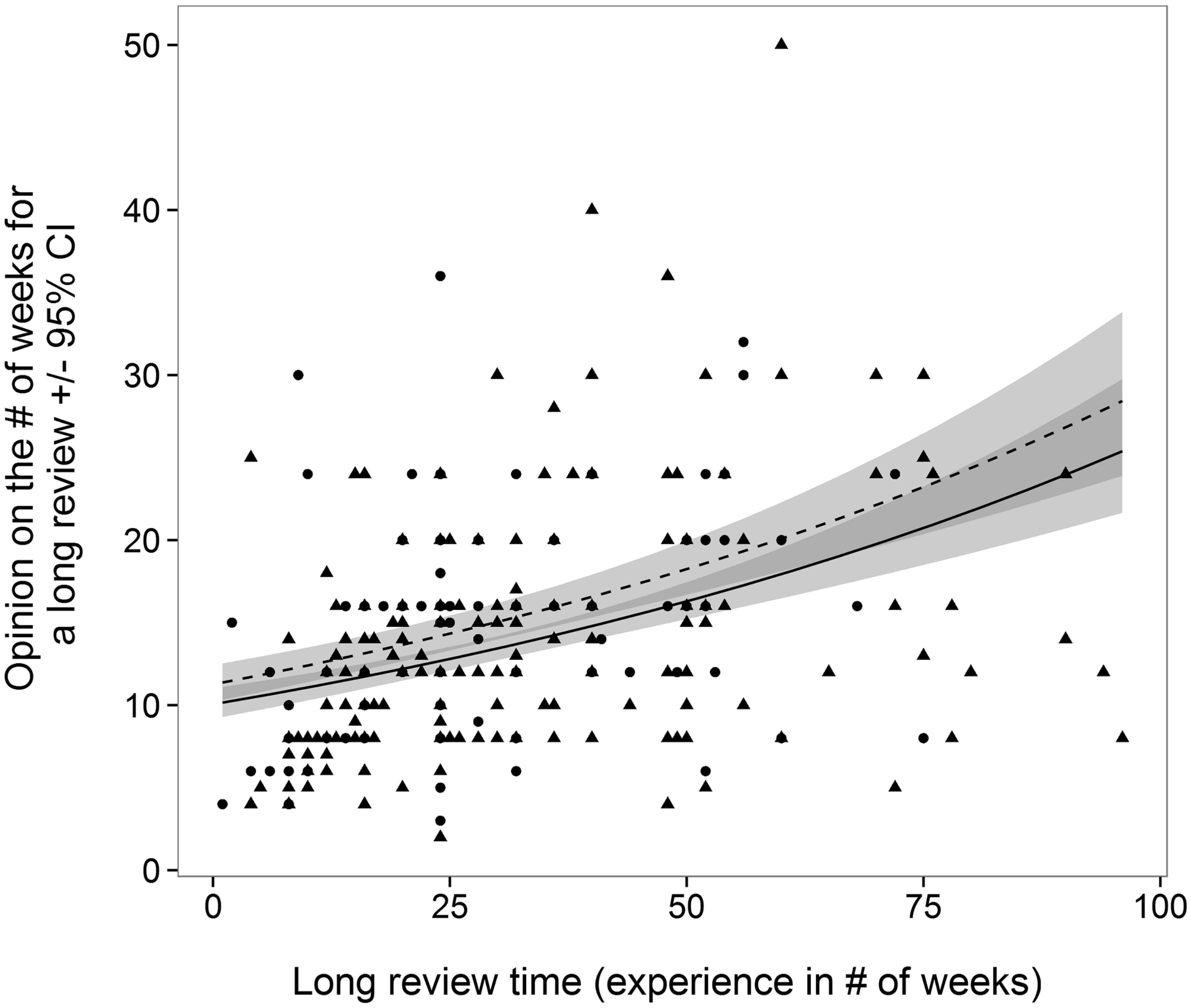

Fig 3. Male (solid line) and female (dashed line) researcher opinion on the time (# weeks ± 95% CI) considered for a long review given their experience (# of weeks for a long review).

doi:10.1371/journal.pone.0132557.g003

less than what was experienced as a "long" review (Fig 3). For example, although a female respondent experienced a long review of 60 weeks, she expects a long review to take just over 20 weeks [18.2, 22.3, 95% CI]. Based on researcher experience and generalizing for all ages, those who identify as male appear to be the least satisfied with the speed of the peer-review process.

Interactions with editors and journals

If a decision has not been made on a manuscript, participants (N = 479) waited on average 12.9 ± 7.5 weeks before making first contact with the editor or journal regarding the status of the manuscript "in review" (Table 1). Of those who make first contact with an editor or journal, most will make additional attempts (77% of 479 responses) if time progresses without a response or decision. Of 479 completed responses, only 9% will never attempt to contact the

Table 2. Frequency (%) of respondents' perspectives on the accountability of the duration of a review process and average score (mode and median) of the Likert type scale with a score of 1 being greatly slows review speed to score of 5 being greatly speeds up review.

Accountability of peer review duration	Greatly slows review speed	Somewhat slows review speed	No impact	Somewhat speeds up review	Greatly speeds up review	Mode	Median
Scientific significance for advancing the field of study (N = 461)	1%	10%	46%	34%	9%	3	3
Conservation implications of results (N = 208)	1%	5%	74%	17%	3%	3	3
Policy implications of results (N = 456)	2%	10%	72%	14%	3%	3	3
Potential public interest or potential for media attention (N = 458)	1%	4%	53%	33%	10%	3	3
Length of paper (N = 462)	12%	55%	29%	3%	1%	2	2
Journal prestige or impact factor (N = 459)	4%	12%	27%	42%	16%	4	4
Maximum 'allocated' review times for each journal (N = 454)	10%	21%	25%	34%	10%	4	3
Persistence of editorial team (N = 460)	3%	10%	18%	44%	25%	4	4
Number of reviewers (N = 464)	22%	58%	13%	7%	2%	2	2
Editor fatigue (lack of time, etc.) (N = 465)	51%	42%	5%	1%	1%	1	1
Reviewer fatigue (lack of time, etc.) (N = 467)	71%	26%	1%	1%	1%	1	1

doi:10.1371/journal.pone.0132557.t002

editor or journal suggesting that the author population in this study is quite proactive in voicing their concerns, but keeping in mind that authors who are proactive are likely to agree to partake in the questionnaire. Nevertheless, this finding is important for editors who may feel confused as to the sort of delays in time before authors begin contacting them. Approximately 12% of participants (N = 469) believed that contacting the editor or journal would jeopardize the decision for acceptance, 6% thought it would benefit the decision, while majority did not believe there was any influence.

Only 14% of respondents (N = 480) have threatened to withdraw their manuscript from the journal, and 15% (of the 480 respondents) have actually withdrawn a submitted manuscript when the review process was unsatisfactorily long, which was indicated to be on average 30 ± 31 weeks (ranging from 2–100 weeks, N = 72) when such actions were deemed necessary. This review duration for a potential withdraw of a manuscript is over double the average time that respondents perceive as slow, indicating that most authors had been quite patient with the peer review process. Despite their apparent patience, respondents generally believe that long reviews should be 300% shorter than what they have experienced (Fig 2), indicating an overall perception that peer-review durations are too slow within the realm of conservation biology.

The majority of participants (72% of 480 responses) did not believe that a long or a short review period would mean that the manuscript was likely to be accepted or rejected. Contrastingly, 14% of respondents believed that a "short" review period would likely lead to a rejection of the manuscript and only 6% believed it would likely be accepted, leaving 8% without opinion. In general, authors did not seem to believe there was any bias toward acceptance or rejection of their manuscript if they contacted the editor or whether the review period was quick or long.

Factors influencing review time and accountability

Of the completed responses (N = 471), over half of the respondents (56%) believed that the reviewers are accountable for the review duration, while 33% held the editors accountable, and 6% attributed delays to the journal staff. The remainder of respondents (5%) believed it was a combination of all the players. Likert type questions revealed that in general, reviewer fatigue (e.g., lack of time, etc.) was ranked as the most influential factor in *slowing* review speed, followed by editor fatigue, and somewhat the length of the manuscript as well as number of reviewers (Table 2). One respondent expressed this reviewer fatigue as follows:

> While editors try to find suitable reviewers in practice there is a relatively small pool of reviewers who can be relied on to do useful reviews. I am an associate editor on 5 journals and am convinced that there is substantial reviewer fatigue out there as the number of publications has grown annually as have the number of journals.

This may correspond with the increased number of publications and publication outlets that contemporary scientists have to contend with. Similarly, in 2007, it was reported that over 1000 new papers appear daily in the scientific and medical literature alone, and this number is likely increasing rapidly [14]. Kumar [25] listed five reasons for publication delay, which included reviewer availability and reviewers having other commitments pushing manuscript reviews at the bottom of their list. The other three reasons included editors sending the manuscript for multiple rounds of reviews (when reviews are conflicting or inadequate); the journal has outsourced manuscript management (e.g., Business Process Outsourcing agency), and; the reviewer intentionally delays the publication of a manuscript for various reasons (e.g., rivalry or intentions to plagiarize).

On the other hand, respondents perceived the persistence of the editorial team as a factor in somewhat speeding up the review process, as well as maximum allocated review times for each journal, and the journal prestige or impact factor (Table 2):

> I will always take the full amount of time they [editors] give me. Moreover, only once have I been asked to review a paper by an open access journal, which required my review submission in 2 weeks. But all the others were non-open access journals that gave me a month or more, which increased the average time to decision.

Consequences of long or short review durations

We questioned participating authors on their perspectives of consequences of long or short review durations. Our findings indicate a number of consequences that we have grouped into themes below.

Consequences for the journals. After a long review period, most respondents (74% of 472 responses) said they are less likely to submit to that journal again relative to other journals; however, some (19%) said it would depend on the journal impact factor or prestige. As expected for the other end, if the review period was short, respondents (69% of N = 471) said they are more likely to submit to that journal again, with some respondents (17%) considering journal impact factor or prestige and 12% of participants were neither more or less likely to submit to that journal again. We also found that review duration is an important factor when respondents (N = 470) consider which journal to submit their research to (43% said yes and 46% said sometimes), while $< 10\%$ of participants said they never consider review duration when submitting a manuscript. Therefore, review time is an important consideration for

journals to maintain reputation, as majority of respondents have given thought to review times when deciding what journal to submit to. Although, there are some indications of trade-offs between review length and impact factor as approx. 1 of 5 respondents consider journal prestige and impact factor as an influential part of deciding to which journal they should submit.

In general, respondents (N = 465) discuss the speed of review with their colleagues, of which 54% (of 465) discuss it monthly, 30% once a year, 12% weekly, 1% daily and 4% never discuss review speed. Interestingly, there was an even split among all respondents (N = 466) with authors (49%) that have "blacklisted" a journal for its lengthy review times (i.e. chosen not to resubmit manuscripts to that journal in the future,) and those who have not (48%). These findings send multiple messages to journal editors: 1) review time is an important factor for authors in consideration of publication outlets, and 2) review time is actively being discussed by half of the respondents, which can hinder or endorse a particular journal's reputation. Publication of research can ultimately affect society at large if the manuscript has significant scientific and policy implications. Therefore, editors/journals/publishers have a responsibility to disseminate scientific information in a timely manner and must play an active role through setting standards and facilitating the peer-review process [25].

Consequences on careers. Just over half of the respondents (55% of 466 respondents) feel that a lengthy peer-review process affects their career, while 30% did not believe it did. Open-ended responses suggested that lengthier peer-review durations generally have negative impacts on "early career researchers" and "young scientists" (mentioned by 65 of 212 responses) because of the "publish or perish" system, which affects opportunities for jobs and career advancement. One respondent wrote:

> As an early career researcher trying to build a list of publications, it is important to have papers reviewed quickly. The longer the time lag between a research project and accepted publication the more difficult it is to apply for new grants or job opportunities.

Furthermore, some respondents mentioned the delay in graduation or acceptance in graduate school for students due to lengthy peer-review processes:

> I received the first response about my first article only after 54 weeks. At that time I was not able to start my PhD because the institution only accepted candidates with at least one accepted article.

> Even after successful completion of my Ph.D. research topic, I was unable to submit my thesis because it's a rule that at the day of Ph.D. thesis submission, must have a minimum one peer reviewed publication.

The comments of these early-career respondents are perhaps reflected in the predictions from Models 2, where despite the length of time they have experienced as a "short" review, respondents consistently expect review periods to be much shorter (Fig 2). It seems that regardless of their experience, the review period cannot be short enough for early-career professionals who publish in conservation biology. In addition, it seems that irrespective of age, respondents believe a lengthy review period should be considerably shorter than what they have experienced (Fig 3).

For respondents with tenure or later in their career, a slow review process can impact applications for grants/funding (approx. 28% of responses) and promotions (approx. 19% of responses):

> Publications are important for ranking of scientists and institution achievements so long reviews and long editorial process could violate this process.

Furthermore, concerns about competition among research groups (5% of responses), subjective treatment, malpractice of certain reviewers and editors, conflicts of interest, and the potential for being "scooped" (i.e., publishing the same idea/findings first) were voiced. Intentional delay of review was also listed as 1 of 5 reasons for peer-review delay by Kumar [25], emphasizing some merit of this topic. Although not the focus of this study, we found that the association between review time and the potential for being "scooped" is worrisome to a number of authors and should be acknowledged as this topic was brought up relatively frequently when respondents were given the opportunity to comment freely (open responses). For example:

> If people play the game well and get their "friends" to review their papers. I am sure in many cases that speeds up the process more so when people cite their friends (the reviewers) in these papers.

> If a person has an "in" with the journal. In other words, subjectivity and preferential treatment increase speed.

Several respondents (<8%) urged that if a manuscript is to be rejected, journals should do so in a timely manner so the researcher can resubmit to another journal sooner. Others voiced concerns that a delay of a manuscript could hinder subsequent work that is built on the manuscript in review, and some mentioned challenges in remembering specifics of the study or content of the manuscript when review times are particularly long.

Consequences to authors' morale. It was also revealed that lengthy peer reviews can affect motivation, causing conflict as well as frustration (8% of responses):

> The frustration associated with a lengthy process discourages the writer. Incentives for conducting research are diminished when rewards are not forthcoming. Less incentive means less motivation which both translate into less productivity. Less productivity means less likelihood for promotions. This in turn sets up a vicious cycle very similar to the one related to applying unsuccessfully for grants.

> A long peer review process reduces drastically your efficiency of publishing papers, because you need to go back to your previous work and you cannot focus on your current work. Sometimes you need to spend quite a bit of time figuring out how to answer reviewer's concerns because it was too long ago that you submitted your manuscript.

> It is very frustrating, and sometimes embarrassing, to have papers endlessly "in review"." I had a paper where the subject editor sat on the paper for 5 months without sending it for review; after 3 contacts they finally sent it for review and it has been another month and we have not heard back. This was a key paper needed to build a grant proposal, and my collaborators consistently asked if it was published yet—the grant was ultimately submitted before the paper was accepted.

These consequences are not often discussed, but are often interlinked with consequences of a researcher's career and aspirations. Although for the majority of the time, long review

durations may not have dramatic consequences; however, lengthy review durations that occur at the wrong place at the wrong time may potentially lead to a cascade of consequences.

Alternative responses to consequences of review times. A number of respondents (<10%) provided interesting alternative responses that are worth mentioning such as (but not limited to) consequences on research quality because of the race to publish, competition among colleagues, greater opportunity cost when taking the time to submit a "quality" manuscript, and limiting peer-review process only to academic research because researchers in other sectors are not rewarded with number of publications and productivity:

> Research quality suffers—as opportunities to publish high quality research can be lost when other groups publish (often lower quality) research first. The focus then becomes speed and simplicity of research rather than quality.

> Because of career pressure, especially for younger scientists, or the need to complete a degree program, choices are often made (I witness them here) to submit smaller, simpler studies to journals with a quick turnaround, or with a presumed higher acceptance rate for a particular work, rather than invest more time in extending analysis and/or facing rejection or extensive revisions

Should the review process be altered?

When asked if respondents thought the review process should be altered to change the review time, 61% (of 463) responded yes, 12% responded no, and the remainder had neutral opinions. Of 462 respondents, 43% believed that the review process should be improved while only 8% said no. We asked how it should be improved, and received 211 open-ended responses from participants (summarized in Table 3).

Referee reward system. About one quarter of the suggestions for improvement was to pay reviewers/editors or provide reviewer incentives/consequences or reward system such as: free year subscription to the journal; rewarding reviewers by adding value to their CV (e.g., "20 best reviews" or "20 best reviewers' awards"); "have a 1 in 2 out policy. . . each paper you submit as a lead author means you have to review 2 for that journal before you can publish again in that journal"; providing discounts on the reviewer's own submissions or items from the scientific publishing house (e.g., books, open access discount, etc.); and home institutions should have reward systems for researchers who regularly review papers.

Table 3. Summary of suggestions to improve the peer review process and increase speed.

Theme	Description	Approximate proportion of responses
Referee reward system	Providing incentives and compensation for reviewers and editors	25%
Deadlines and defined policies	Shorted allocated time to review a manuscript and strict procedures to ensure adherence to deadlines	30%
Editorial persistence	Proactivity from editors to send reminders, follow up with deadlines, and setting the tone.	14%
Improved journal management	Overall management of editorial staff and inter-journal management	6%
Change norms of publishing	Author empowerment, journal standardization, open peer review, double blind reviews	12%
Alternative responses	Permitting to submit to more than one journal, include early career researchers as reviewers, follow model of journals that do it well, bank or database of reviewers, have sub-reviewers (e.g. expertise for statistics, methods, taxa, tools, etc.)	13%

doi:10.1371/journal.pone.0132557.t003

> Editors should remove slow reviewers from their lists. There should be a central bank where good reviewers receive benefits such as fast track review of their material if submitted to the same company (e.g. Wiley, Elsevier, etc.). A reduction in publication costs for good reviewers (not just time but quality of revision)

> Engagement for reviewing should be better acknowledged as a performance indicator; some exemplary review processes should be made public so that authors and reviewers can learn from them. Reviewers should be able to see the other reviewer's comments after the editor's decision.

> For instance, the journal Molecular Ecology is publishing the list of the best reviewers every years based on the quality and speed of the review. This is one example of a rewards that the reviewers can put in their CV to show their importance in the field.

Our findings suggest there is some weighted call for reviewer incentives and reward systems. The editor of BMJ, Richard Smith [26], estimated the average cost of peer review per paper was approximately $US 150 (keeping in mind 60% are rejected without external review), whereas the cost of papers that made it to review was closer to $US 1500 and without considering opportunity costs. A recent survey reported two-thirds of academics agreed that $100–200 would motivate them for reviewing while one-third refused to accept monetary compensation [27]. Kumar [25] reports differing results from two recent studies where one study of 1500 peer reviewers in the field of economics responded to both monetary and non-monetary incentives to expedite the return of reports [28], while in 2013, Squazzoni et al. [29] reported that financial incentives decreased the quality and efficiency of peer reviewers.

Reward system and incentives for reviewers have been proposed in the literature [30], where there may be penalties to those who decline reviews or non-monetary rewards for review completions such as published lists of reviewers as a means of acknowledgment (e.g. Journal of Ecosystems and Management). However, some journals already use this system and still there is no indication of change in referee behavior [31]. One common incentive given for peer-review is a temporary subscription to the journal in question. It is perhaps not surprising that such an incentive might fail to change reviewer behavior, since many reviewers will belong to institutions that already possess subscriptions to a host of journals

It may just be a matter of time for the “top reviewers” or time spent on reviews to become “prestigious” and valued in more tangible ways (whereas current system values number of publication). Peerage for Science is a novel approach to externalized peer-review, through which manuscripts are submitted for peer-review by members of a community of researches in a transparent and non-blinded way, after which journals can then be contacted with an amended draft [32]. This system incentivizes peer-reviewers by providing metrics and ratings relating to their reviewing activities that members can use to demonstrate their activities.

Deadlines and defined policies. Approximately one third of responses (N = 211) suggested stricter deadlines and policies, shorter allocated time to review a manuscript, and procedures to ensure adherence to strict deadlines should be established to improve review duration:

> Current review process should follow the model of the PLOS (online journals). Reviewers are constrained to address specific scientific elements: The question, the method, the results and the discussion that these are scientifically acceptable. This should encourage young researchers to publish without the need to include big names/ popular personalities in research to have the paper through journal review.

Again improvements in peer review turnaround and quality are something that the journal editors are able to control by setting out standards and policies that facilitate the peer review process. A recent review of time management for manuscript peer-review acknowledged several suggestions to improve the review process and time, but that it is the responsibility of editors, publishers and academic sponsors of the journals to implement these improvements [25].

Editorial persistence and journal management. Related to these more stringent deadlines and policies is the suggestion that editors should put more pressure on reviewers, and follow up with deadlines (30 responses), while others suggested better journal management (13 responses):

> Some Journals restart the time counting during a revision process, for example, asking to resubmit as a new manuscript in order to reduce the revision time, instead of keeping track of the time during the whole revision process and to be more realistic about the time that a revision takes. I believe that is a way of cheating or deceiving the system.

As illustrated by the quote above, many journals ask to re-submit as a "new submission" rather than a "resubmission", and sends to new referees instead of the previous ones to review the revisions, which increases the length of peer review time. Fox and Petchey [31] suggested that if a manuscript is rejected from one journal, the reviews should be carried forward to the subsequent journal that the manuscript was submitted to. They argued this action helps with quality control and facilitate review process by ensuring that authors revise their manuscripts appropriately, and reduces any duplication of efforts by referees. At present, at least one ecology journal allows authors of manuscripts previously rejected to provide previous reviews and the publisher Wiley is trialing peer-review transfer across nine of its neuroscience journals [33]. A more formal system for sharing reviews is suggested to increase speed and quality of the peer review system, which is now feasible with the pervasive use of electronic submission and review systems [31].

Peer Review training. Including graduate students or early career researchers as reviewers may increase the "supply" for the increasing demand. Some may argue that graduate students lack experience and knowledge to appropriately assess a manuscript. Formal training has been suggested to improve quality of reviews and increase the network of reviewers. Furthermore, recommendations by senior researchers of names of reliable and qualified graduate students or early career researchers as potential reviewers may help with the deficit [32]. Indeed, the British Ecological Society recommends that academic supervisors should assign their own peer-review invitations to graduate students [34], although it is certainly sensible to verify that individual journal editors are happy with this practice.

Changes to the norms of peer-review system. A number of respondents (12%) wanted to see more drastic changes in the norms of publishing. For example, permanent and paid group of reviewers, standardizing all journals, permitting to submit manuscripts to more than one journal, including more early career researchers as reviewers, following model journals that do it well (e.g., Geoscience, PLOS one), having a database of reviewers, or have sub-reviewers (e.g. expertise for statistics, methods, taxa, tools, etc.).

"PubCreds" currency, has been proposed as a system where reviewers "pay" for their submission using PubCreds they have earned by performing reviews [31]. Although, a radical idea, Fox and Petchey [31] state that "doing nothing will lead to a system in which external review becomes a thing of the past, decision-making by journals is correspondingly stochastic, and the most selfish among us are the most rewarded". Furthermore, Smith [26] suggested adopting a "quick and light" form of peer review, with the aim of opening the peer-review system to the broader world to critique the paper or even rank it in the way that Amazon and other retailers

ask users to rank their products. Alternatively, some journals (e.g. Biogeosciences) employ a two-stage peer-review, whereby articles are published in a *discussions* format that is open to public review prior to final publication of an amended version. Other journals (e.g. PLOS ONE) and platforms (www.PubPeer.com) offer the opportunity for continued review following publication. The argument for a radical change in the norms is not uncommon and may be required in today's peer-review system which will soon be in crisis (31).

Role of open access and journal prestige on review duration. The majority of respondents do not review a manuscript quicker for higher tier journals (71% of 445 respondents). When respondents were asked about their perception on the justification of journal prestige on turnaround time, 50% of 369 responses do not believe publishing in a top-tier journal justifies a rapid or delayed review time, while 37% believe it does (remainder had no opinion). Of those who believed publishing in a top-tier journal justifies longer or shorter review time, 64% believe it explains rapid reviews, 14% believe it justifies a delayed review, and 20% believe it justifies both rapid and delay (<5% believe neither). On the other hand, it was interesting to note that a higher number of respondents (75% of 367) believe that publishing in a low-tier journal does not justify a rapid or delayed review time. Overall, journal prestige and impact factor seem to be an important indicator for many authors, although their ability to turnaround peer-review in a timelier manner may reflect their perceived prestige and the higher quality manuscripts that make it through primary editorial screening.

We were interested in participants' perspectives of the review process for open access (OA) journals, particularly because authors pay a fee to publish in such journals. About a third (32% of 461) agreed that OA journals should have higher quality of "customer service", such as faster review and publication times, with an additional 13% of respondents who strongly agree. Another third (31%) of respondents were neutral about this statement, whereas 16% disagree and 7% *strongly* disagree. This finding is interesting because it is unlikely that a journal's publishing standards will change as a result of a switch to OA; indeed the editorial and peer-review standards should be entirely disconnected from cost. It is strange then that a large proportion of respondents expect higher quality from OA journals. This is most likely the result of a shift in the customer base. In subscription-based publishing the customer is the librarian and their measure of product quality was assessed primarily through metrics such as Impact Factor. In OA publishing, the customer becomes the submitting researcher, and quality is assessed through publishing service and, incorrectly perhaps, standards of editorial review. It has yet to be proven that publishers will see substantial increases in profits following a switch to OA, and if profit margins are not significantly increased then expectations of improved service are unwarranted.

Although the topic of open access journals was not the primary focus of our study, we believe that it is an increasing relevant topic as there are debates about the quality of OA journals, but on the other hand, open access may be viewed as mandatory, particularly where research is funded with public money. Future research including perspectives and understanding value of OA journals within the conservation science community should be considered.

> There is likely a link between review duration and impact factors, as impact factors are based on citations during the first two years after publication. If those citing papers take longer to go through the review, they won't count towards the journal's impact factor.

> The quality of review should have nothing to do with "impact" factors. These are biased indices with no measured reality against which they are measured. They are also self-reinforcing. An honest scientist should review each paper on its merit.

Synthesis

Our findings show that the peer-review process within "conservation biology" is perceived by participating authors to be slow (14 weeks), and turnaround times that are over double the length of what they perceive as "optimal" (6 weeks). In particular, males seem to expect shorter review times than females, whereas female expectations were found to be more closely related to what they have actually experienced in typical review times. Similarly, older participants (> 40 years) have expectations of review times that are more closely aligned with their experience, while younger authors developed their opinion of a short review time to be <10 weeks despite their experiences. Overall, the primary reasons that participants attribute to the lengthy peer-review process is the "stress" on the peer review system, mainly reviewer and editor fatigue. Meanwhile, editor persistence and journal prestige/impact factor were believed to speed up the review process. The institutional incentive for productivity has its fallacies. The demand from increased publications strains the peer-review system and the "publish or perish" environment can also potentially create a strong demand for publications outlets and increased expectations for quick turnaround times.

It appears that early career researchers are more vulnerable to slow peer review durations in a "publish or perish" system as it relates to graduation employment opportunities and other career advancements. Closely related to impacts on careers are consequences of lengthy peer review duration on an author's "morale" (i.e. motivation, frustration, conflicts, embarrassment). Some respondents commented that lengthy review durations may result in lack of motivation, forgotten details about the manuscript thus leading to reduced efficiency in productivity and potentially a lower quality manuscript. Competition among colleagues was thought by few respondents to encourage publication of shorter and simpler studies in order to gain a quicker turnaround review time, rather than investing more time in complex and extensive analyses or revisions. These concerns have merit as they do exist and may have implications on quality of research and publications.

Although the objective of our research was not to assess the quality of the peer-review system, we believe all aspects of the process are interlinked and both peer review quality and speed are not mutually exclusive and must be discussed simultaneously. The majority (61%) of respondents believe that the review process should be altered with a number of suggestions such as a referee reward system, defined deadlines and policies, editorial persistence, better journal management, changing the norms of the peer-review process and others. Currently, researchers are rewarded based on productivity, which may result in a system breakdown by increasing demand from a short supply of reviewers and subsequently degrading quality of publications associated with the race to publish (34). We suggest a partial shift in institutional rewards and incentives from researcher productivity to greater outreach efforts and public interactions/activities, as there is evidence that conservation goals may be more effectively achieved by engaging the public. Implementing a system that rewards these actions in conjunction with productivity may alleviate pressure in the peer review system overall, and increase conservation successes. Training for peer review is a possibility to improve quality of reviews as well as increase the pool of reviewers by including early career scientists and graduate students. Generally, there is a call from a number of authors to revise and review our own peer review system to ensure its persistence and quality control.

Open access and opening the peer review process is on the forefront of publishing innovation. For example, PeerJ (www.peerj.com) offers a novel approach that combines open access and a pre-print system that enables articles to be made available online more rapidly than traditional scholarly publishing. Such approaches will require time to determine their value to the scientific community, but as scholarly publishing continues to rapidly evolve, experimental

approaches to enhancing the communication of peer-reviewed research are warranted. We encourage other scientists and publishers to build on these approaches and continue to push the envelope for new publishing approaches.

Conclusion

Peer reviewed journals will continue to be the primary means by which we vet scientific research and communicate novel discoveries to fellow scientists and the community at large, but as shown here, there is much room for improvement. We provided one of the first evaluations of an important component of the publishing machine, and our results indicate a desire for researchers to streamline the peer-review process. While our sample may not be generalizable to the global community of researchers submitting papers in the realm of conservation biology, we believe the opinions, perceptions, and information provided here present an important collective voice that should be discussed more broadly. While the technology is in place to accelerate peer-review, the process itself is still lagging behind the need of researchers, managers, policy-makers, and the public, particularly for time-sensitive research areas such as conservation science. Moving forward, we should encourage experimental and innovative approaches to enhance and expedite the peer-review process.

Supporting Information

S1 File. Complete list of survey questions.
(DOCX)

S2 File. GLM data analysis supplement for Models 1–3.
(DOCX)

S3 File. Raw questionnaire data.
(XLSX)

Acknowledgments

We thank all of the study participants who took the time to share their perspectives. Funding was provided by the Canada Research Chairs Program and the Natural Sciences and Engineering Research Council of Canada.

Author Contributions

Conceived and designed the experiments: SJC NH AJG MRD NRH ADMW VMN. Performed the experiments: VMN LFGG NRH. Analyzed the data: VMN LFGG. Contributed reagents/materials/analysis tools: VMN LFGG SJC. Wrote the paper: VMN NRH LFGG ADMW AJG MRD NH SJC.

References

1. Gilbert GN (1976) The transformation of research findings into scientific knowledge. Social Studies of Science 6: 281–306.
2. Robertson M (2009) What are journals for? Journal of Biology 8: 1.
3. Smith R (2006) Peer review: a flawed process at the heart of science and journals. Journal of the Royal Society of Medicine 99(4): 178–182. PMID: 16574968
4. Bohannon J (2013) Who's afraid of peer review? Science 342: 60–65 doi: 10.1126/science.342.6154.60 PMID: 24092725
5. Tregenza T (2002) Gender bias in the refereeing process? Trends in Ecology & Evolution, 17(8), 349–350.

6. Clavero M (2011) Language bias in ecological journals. Frontiers in Ecology and the Environment doi: 10.1890/11.WB.001
7. Primack RB, Ellwood E, Miler-Rushing AJ, Marrs R, Mulligan A (2009) Do gender, nationality, or academic age affect review decisions? An analysis of submissions to the journal Biological Conservation. Biological Conservation 42: 2415–2418.
8. Budden AE (2008) Double-blind review favours increased representation of female authors. Trends in Ecology and Evolution 23:4–6. PMID: 17963996
9. Sih A, Ferrari MC, Harris DJ (2011) Evolution and behavioural responses to human induced rapid environmental change. Evolutionary Applications 4(2): 367–387 doi: 10.1111/j.1752-4571.2010.00166.x PMID: 25567979
10. Steffen W, Crutzen PJ, McNeil Jr (2007) The Anthropocene: are humans now overwhelming the great forces of nature. Ambio: A Journal of the Human Environment 36 (8): 614–621.
11. Sutherland WJ, Pullin AS, Dolman PM, Knight TM (2004) The need for evidence-based conservation. Trends in Ecology & Evolution 19(6): 305–308.
12. Harnad S (1996) Implementing peer review on the Net: scientific quality control in scholarly electronic journals. In Peek R. and Newby G., eds. Scholarly publishing: the electronic frontier. MIT Press, Cambridge, MA.
13. Kim HJ (2001) The transition from paper to electronic journals: key factors that affect scholars' acceptance of electronic journals. The Serials Librarian 41(1): 31–64.
14. Grainger DW (2007) Peer review as professional responsibility: A quality control system only as good as the participants. Biomaterials 28: 5199–5203. PMID: 17643484
15. Schäfer RB, Cooke SJ, Arlinghaus R, Bonada N, Brischoux F, Casper AF et al. (2011) Early career researchers' perspectives on the current and future state of the scientific publication process in ecology. Freshwater Biology 56: 2405–2412.
16. Soulé ME (1985) What is conservation biology? BioScience 35, 727–734.
17. Jefferson T, Wager E, Davidoff F (2002) Measuring the quality of editorial peer review. Jama, 287(21): 2786–2790. PMID: 12038912
18. Ng KH (2009) Exploring new frontiers of electronic publishing in biomedical science. Singapore Medical Journal 50(3): 230–234. PMID: 19352562
19. Wood D. (1998). Online peer review? Learned Publishing 11(3): 193–198.
20. Tananbaum G, Holmes L (2008) The evolution of web-based peer-review systems. Learned Publishing 21 (4): 300–306.
21. Strauss AL. Basics of qualitative research: techniques and procedures for developing grounded theory 2nd ed. SAGE Publications Inc. Thousand Oaks, United States; 1998.
22. Chambers JM (1992) Linear models. Chapter 4 of Statistical Models in *S* eds Chambers J. M. and Hastie T.J., Wadsworth & Brooks/Cole
23. Zuur AF, Ieno EN, Walker N, Saveliev AA, Smith GM (2009) Mixed effects models and extensions in ecology with R. New York: Springer.
24. Mulligan A, Hall L, Raphael E (2013) Peer review in a changing world: An international study measuring the attitudes of researchers. Journal of the American Society for Information Science and Technology 64(1): 132–161.
25. Kumar MN (2014) Review of the Ethics and Etiquettes of Time Management of Manuscript Peer Review. Journal of Academic Ethics 12(4): 333–346.
26. Smith R (2006) Peer review: a flawed process at the heart of science and journals. Journal of the Royal Society of Medicine 99: 178–182. PMID: 16574968
27. Davis P. (2013; Internet). Society for Scholarly Publishing—Rewarding reviewers: money, prestige, or some of both? [updated 2013 Feb 22] Available: http://scholarlykitchen.sspnet.org/2013/02/22/rewarding-reviewers-money-prestige-or-some-of-both/. Accessed 27 February 2015.
28. Chetty R, Saez E, Sándor L (2014) What policies increase prosocial behavior? An experiment with referees at the Journal of Public Economics. Journal of Economic Perspectives 28: 169–188.
29. Squazzoni F, Giangiacomo B, Károly T (2013) Does incentive provision increase the quality of peer-review? An experimental study. Research Policy 42(1): 287–294.
30. Hauser M, Fehr E (2007) An incentive solution to the peer review problem. PLOS Biology doi: 10.1371/journal.pbio.0050107
31. Fox J, Petchey OL (2010) Pubcreds: fixing the peer review process by "privatizing" the reviewer commons. Bulletin of the Ecological Society of America 91 (3): 325–333.

32. Hettyey A, Griggio M, Mann M, Raveh S, Schaedelin FC, Thonhauser KE et al. (2012) Peerage of Science: will it work? Trends in Ecology and Evolution 27(4): 189. doi: 10.1016/j.tree.2012.01.005 PMID: 22325345

33. Wiley Online Library [Internet]. Transferable Peer Review Pilot. Available: http://olabout.wiley.com/WileyCDA/Section/id-819213.html. Accessed 27 February 2015.

34. British Ecological Society [Internet]. A guide to peer review in ecology and evolution. Available: http://www.britishecologicalsociety.org/wp-. Accessed 27 February 2015.
